# The impact of starchy food structure on postprandial glycemic response and appetite: a systematic review with meta-analysis of randomized crossover trials

**DOI:** 10.1093/ajcn/nqab098

**Published:** 2021-05-28

**Authors:** Mingzhu Cai, Bowen Dou, Jennifer E Pugh, Aaron M Lett, Gary S Frost

**Affiliations:** Section for Nutrition Research, Department of Metabolism, Digestion and Reproduction, Faculty of Medicine, Imperial College London, Hammersmith Campus, London, United Kingdom; Section for Nutrition Research, Department of Metabolism, Digestion and Reproduction, Faculty of Medicine, Imperial College London, Hammersmith Campus, London, United Kingdom; Section for Nutrition Research, Department of Metabolism, Digestion and Reproduction, Faculty of Medicine, Imperial College London, Hammersmith Campus, London, United Kingdom; Section for Nutrition Research, Department of Metabolism, Digestion and Reproduction, Faculty of Medicine, Imperial College London, Hammersmith Campus, London, United Kingdom; Section for Nutrition Research, Department of Metabolism, Digestion and Reproduction, Faculty of Medicine, Imperial College London, Hammersmith Campus, London, United Kingdom

**Keywords:** starchy foods, food structure, postprandial, glucose, insulin, appetite, randomized crossover trials

## Abstract

**Background:**

Starchy foods can have a profound effect on metabolism. The structural properties of starchy foods can affect their digestibility and postprandial metabolic responses, which in the long term may be associated with the risk of type 2 diabetes and obesity.

**Objectives:**

This systematic review sought to evaluate the clinical evidence regarding the impact of the microstructures within starchy foods on postprandial glucose and insulin responses alongside appetite regulation.

**Methods:**

A systematic search was performed in the PUBMED, Ovid Medicine, EMBASE, and Google Scholar databases for data published up to 18 January 2021. Data were extracted by 3 independent reviewers from randomized crossover trials (RCTs) that investigated the effect of microstructural factors on postprandial glucose, insulin, appetite-regulating hormone responses, and subjective satiety scores in healthy participants.

**Results:**

We identified 745 potential articles, and 25 RCTs (*n* = 369 participants) met our inclusion criteria: 6 evaluated the amylose-to-amylopectin ratio, 6 evaluated the degree of starch gelatinization, 2 evaluated the degree of starch retrogradation, 1 studied starch–protein interactions, and 12 investigated cell and tissue structures. Meta-analyses showed that significant reductions in postprandial glucose and insulin levels was caused by starch with a high amylose content [standardized mean difference (SMD) = −0.64 mmol/L*min (95% CI: −0.83 to −0.46) and SMD = −0.81 pmol/L*min (95% CI: −1.07 to −0.55), respectively], less-gelatinized starch [SMD = −0.54 mmol/L*min (95% CI: −0.75 to −0.34) and SMD = −0.48 pmol/L*min (95% CI: −0.75 to −0.21), respectively], retrograded starch (for glucose incremental AUC; SMD = −0.46 pmol/L*min; 95% CI: −0.80 to −0.12), and intact and large particles [SMD = −0.43 mmol/L*min (95% CI: −0.58 to −0.28) and SMD = −0.63 pmol/L*min (95% CI: −0.86 to −0.40), respectively]. All analyses showed minor or moderate heterogeneity (I^2^ < 50%). Sufficient evidence was not found to suggest how these structural factors influence appetite.

**Conclusions:**

The manipulation of microstructures in starchy food may be an effective way to improve postprandial glycemia and insulinemia in the healthy population. The protocol for this systematic review and meta-analysis was registered in the international prospective register of systematic reviews (PROSPERO) as CRD42020190873.

## Introduction

Type 2 diabetes (T2DM) and obesity produce the greatest global burden on public health services worldwide (1). Global rates of obesity have surpassed 300 million (2). Concurrently, T2DM affects 460 million people worldwide (3). High-glycemic foods play a significant role in the increased incidence of T2MD and development of obesity (4, 5).

Starch is a carbohydrate that accounts for a significant proportion of global nutrient intake. Glycemic responses (GRs) to starchy foods depend on the rate and extent of digestion in the small intestine. Starch is classified into rapidly digested starch (RDS), slowly digested starch (SDS), and resistant starch (RS) (9). RDS evokes a high GR (10), while SDS is steadily absorbed as glucose, attenuating the postprandial GR (11). Several studies have associated a higher SDS intake with increased satiety and lower reduced body weight (12). RS is starch that cannot be digested in the small intestine and reaches the colon for fermentation by gut microbiota. SCFAs, derivatives of fermentation, trigger release of anorectic gut hormones [peptide tyrosine–tyrosine (PYY) and glucagon-like peptide 1 (GLP-1)], promoting satiety (13). Acute feeding studies have shown RS improves the postprandial glycemic response and appetite control (14).

Food structure regulates the rate and extent of starch digestion. Food structure is the arrangement of food constituents at multiple-length scales (15), whether formed naturally, by food processing, or both (16). Food macrostructure refers to structures visible to the eye. Microstructure is the organization of food constituents at the microscopic level (<100 µm) (15).

**FIGURE 1:**
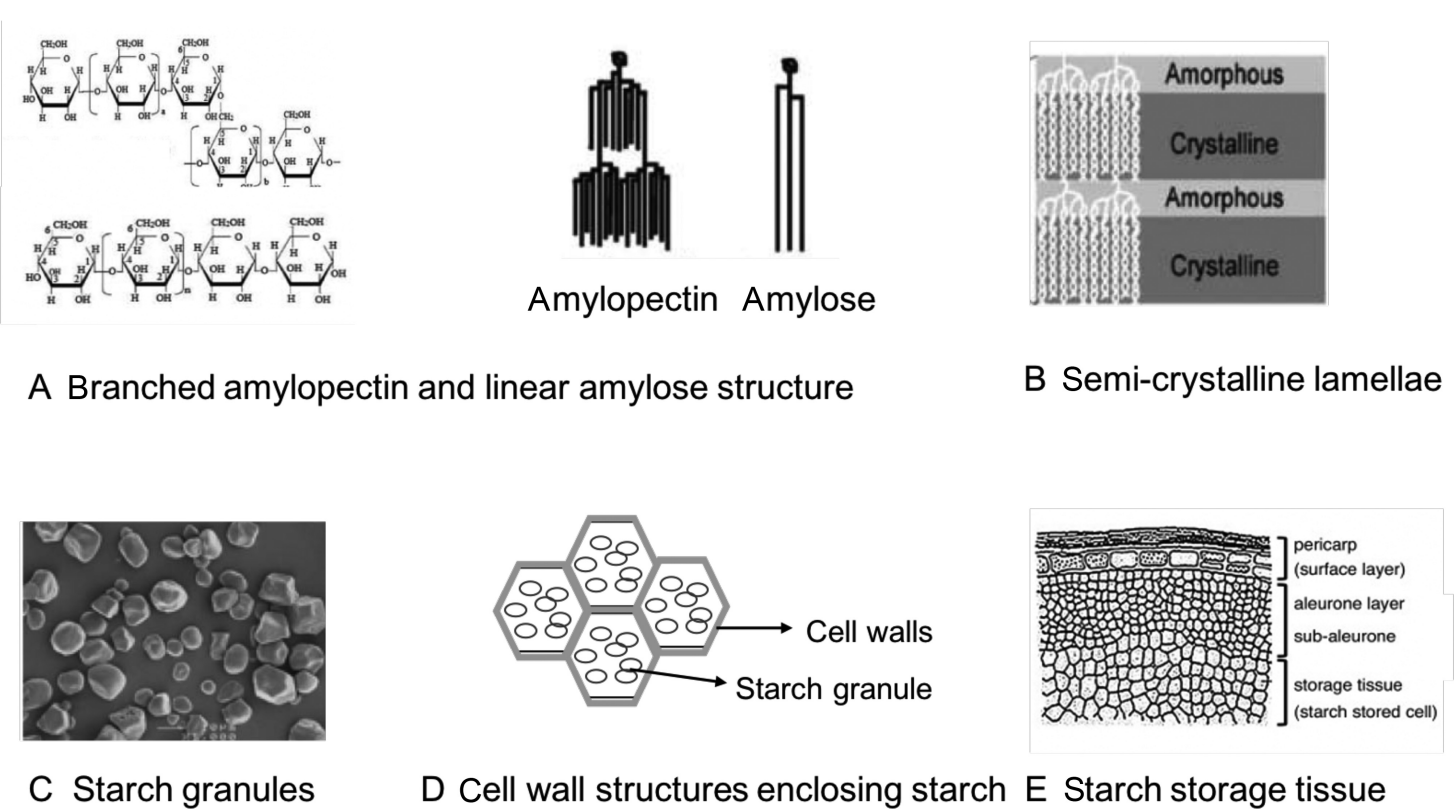
Hierarchical microstructures that can control the rate and extent of starch digestibility. Figure adapted and modified from Tran et al. (6), Tian et al. (7), and Ogawa et al. (8).

Starch, at a molecular level, exists as 2 forms of glucose polymers: amylose and amylopectin (**Figure 1**A), which form the semi-crystalline starch granule (Figure 1B and C). Starch granules, alongside proteins and lipids, are embedded in cell wall structures (Figure 1D). The ratio of amylose to amylopectin (17), morphology of the starch granule (18, 6), starch–lipid interactions (19), and starch–protein interactions (20) influence digestibility. Variations in thickness and permeability determine a cell wall's capacity to limit digestive enzyme penetration into and carbohydrate diffusion out of cells (7, 21).

Many starchy foods undergo processing before consumption, including particle size reduction (mechanical processing or mastication), thermal treatment, and storage (22). Particle size is directly associated with cell wall rupture (23, 24). Smaller food particles tend to have lower cellular integrity and a larger surface area for enzyme action, resulting in greater starch digestibility than larger particles (24, 25, 8). The combination of heat and water promotes starch gelatinization, whereby granules swell, losing their molecular organization. Gelatinization is positively associated with starch digestibility (26). Gelatinized starch retrogrades when refrigerated. Retrograded structures are not recognized by enzymes and are less digestible (27).

Starch digestibility can be controlled through structural changes at molecular, starch granular, cellular, and food processing levels (7); however, their effects upon metabolic outcomes have yet to be reviewed. This systematic review and meta-analysis aims to assess the value of targeting the food structure at each level as a strategy toward promoting desirable metabolic responses to lower rates of T2DM and obesity.

## Methods

This systematic review was conducted in accordance with the Preferred Reporting Items for Systematic Reviews and Meta-Analyses (PRISMA) Statement (28). The review was prospectively registered on a Systematic Literature Review registration website, PROSPERO, as CRD42020190873.

### Eligibility criteria

The PICOS (patients, intervention, comparator, outcome, study design) criteria were used to establish study eligibility and focus the research question (**Table 1**).

**TABLE 1:** PICOS criteria for inclusion and exclusion of studies

Parameter	Criteria	Exclusion
Population	Healthy adults	Animals
Intervention	Consumption of starchy foods with limited starch digestibility caused by their microstructural properties	—
Comparator	Consumption of starchy foods with higher starch digestibility caused by their microstructural properties	Unmatched energy or macronutrients intake (difference > 10%)
Outcome	Acute postprandial glycemic response, gut hormone response, and appetite	Studies which do not contain these outcomes of interest
Study design	RCTs	Not RCTs

Abbreviations: PICOS, patients, intervention, comparator, outcome, study design; RCT, randomized crossover trial.

### Search strategy

Research literature databases PubMed, Embase, Ovid Medicine, and Google Scholar were searched for peer-reviewed articles published up to 18 January 2021. The PubMed database was searched for the following combination of terms: blood glucose[MeSH Terms] OR glycemic index[MeSH Terms] OR insulin[MeSH Terms] OR C-Peptide[MeSH Terms] OR appetite regulation[MeSH Terms] OR appetite regulating hormone[MeSH Terms] OR Peptide YY[MeSH Terms] OR Glucagon-Like Peptide 1[MeSH Terms] OR energy intake OR satiety response[MeSH Terms] AND (human[MeSH Terms] OR adult[MeSH Terms] OR health, women s[MeSH Terms] OR health, men s[MeSH Terms]) AND (starch[MeSH Terms]) OR carbohydrate[MeSH Terms]) AND “diet”) AND trials, randomized clinical[MeSH Terms]. The search strategies for other databases are presented in **Supplemental Table 1**.

### Study selection

All articles identified by the search strategy were imported to Endnote, which was used to eliminate duplicated articles. All articles were reviewed by 3 reviewers independently (MC, BD, and JEP). In the first pass, article's titles and abstracts were screened to determine their possible suitability for inclusion. Selected studies then underwent full-text screening by MC, BD, and JEP independently. For both screening and assessments of study eligibility, disagreements as to the suitability of certain papers were resolved by either consulting a third party (AML) or by discussion until a consensus was reached.

### Risk of bias assessment

The risk of bias (RoB) within eligible studies was independently assessed by 3 authors (MC, BD, and JEP) using the Cochrane RoB2 tool (29). This tool identifies the level of RoB (low risk, some concerns, and high risk) on 5 domains, including the randomization process, deviations from intended intervention, missing outcome data, measurement of outcomes, and selection of the reported result. Studies that were judged to be at low RoB for all domains were considered to have an overall low risk. Studies that were judged to raise some concerns in at least 1 domain but not to be at high RoB for any domain were classified as having some concerns. Studies that were judged to be at high RoB for at least 1 domain were classified as high risk, and studies judged to have some concern of RoB in multiple domains were also labeled as high risk, since multiple concerns may substantially reduce the credibility of the results. Inconsistencies between authors’ RoB assessments at the study level were resolved through discussion until reaching a consensus.

### Data extraction

Upon completion of eligibility screening and RoB assessments, data were independently extracted from each eligible article by 3 authors (MC, BD, and JEP). Data collected included a reference (authors, year of publication), study design and level of blindness, participant characteristics (population, sex, health status, age, and BMI), and intervention and control (test foods, grams of carbohydrate, outcomes of interest (postprandial glucose response, gut hormone response, and subjective satiety score).

Demographic data and described outcome values were extracted as standardized mean differences (SMDs) ± SEs between intervention and control groups. The GR was reflected as blood glucose and insulin incremental AUCs (iAUCs). Appetite was measured by a subjective appetite score iAUC, using a visual analogue scale, and an appetite-regulating hormone response iAUC. These iAUCs were calculated using the trapezoidal rule. The averages of fasting measurements were used as baseline values, and areas below baseline were subtracted. When iAUCs for multiple periods were reported, the iAUC_0–120min_ was included, as it is a dynamic representation of postprandial GR to a carbohydrate-rich meal, which is the primary outcome in this review. Missing data were obtained either by contacting the original investigators or extracting from the figures using a web-based plot-digitizing tool (WebPlotDigitizer) (30). When multiple intervention and control groups (>2) existed in 1 study, all relevant intervention groups were combined into a single intervention group, and all relevant control groups were combined into a single control group. A single pair-wise comparison was created by calculating the combined mean and SD based on the formulas in the Cochrane Handbook (31).

### Calculation of summary measures

Effect sizes and variances for each randomized crossover trial (RCT) were calculated in accordance with the Cochrane Handbook (31). When studies did not report the SDs for paired differences, SDs were calculated from available statistics (e.g., *P* values or *t* statistics). When these statistics were lacking, the SD was estimated assuming a correlation at a conservative level of 0.5 between intervention and control groups to approximate the paired analyses.

### Data analysis

Review Manager version 5.3 (the Cochrane Collaboration, Software Update) was used for random effect model meta-analyses. SMDs with 95% CIs for continuous outcomes (iAUCs for glucose, insulin, gut hormone, and satiety score) were assessed. Heterogeneity was quantified with the I² statistic. An I² value greater than 50% represents significant heterogeneity. When heterogeneity was significant, a sensitivity analysis was conducted to detect the influence of a single study on the overall estimate. A meta-analysis was performed in cases where at least 2 studies were included for each characteristic. A *P* value of < 0.05 was considered to be statistically significant.

### Additional analyses

Sensitivity analyses were performed to determine whether the overall results were affected by imputing different correlation coefficient factors (0, 0.25, 0.75) to approximate paired analyses.

## Results

### Identified trials

**FIGURE 2:**
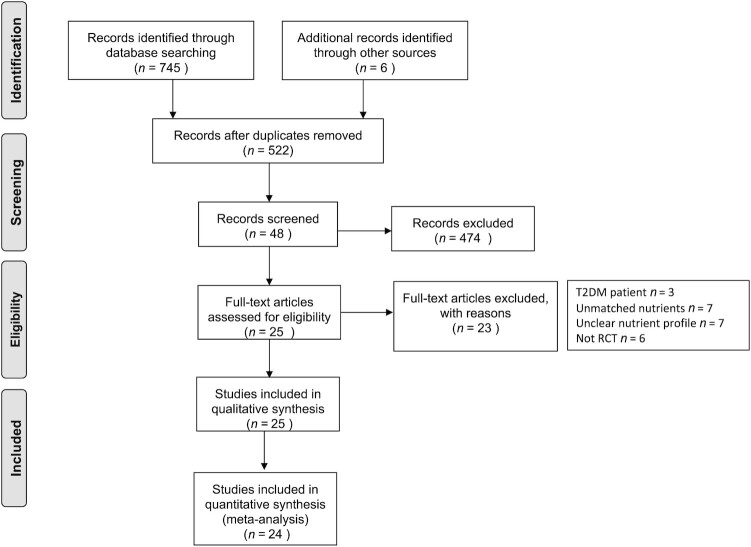
PRISMA flow diagram of the literature search and screening process. Abbreviation: PRISMA, Preferred Reporting Items for Systematic Reviews and Meta Analyses; RCT, randomized controlled trial; T2DM, type 2 diabetes.

A total of 745 articles were identified by the conducted search strategy. Of these, 522 remained after removing duplicates. The initial screening for title and abstract excluded 474 articles that were not relevant to the topic. Of the remaining 48 records, 25 were excluded due to unhealthy populations (*n* = 3), unmatched macronutrient intakes (*n* = 7), unclear nutrient profiles (*n* = 7), and inappropriate study methods (not an RCT; *n* = 6). A total of 25 articles were eligible and included in this systematic review. The literature search and screening process are presented in **Figure 2**.

A total of 369 healthy subjects, aged from 18 to 70 years, were investigated in the 25 included trials. The mean participant BMI ranged from 20.2 kg/m^2^ to 28.8 kg/m^2^. The year of published articles ranged from 1983 to 2020. Studies were grouped according to the microstructures used in the intervention, including the amylose-to-amylopectin ratio (32–37), degree of gelatinization of starch (38–43), degree of retrogradation of starch (32, 44), starch–protein interaction (45), and cell and tissue structures (37, 41, 46–56). More detailed characteristics of the included studies are listed in **Table 2**.

**TABLE 2:** Characteristics of included studies (*n* = 25 RCTs)

Reference	Study design	Participants (health status, number of participants, sex, BMI, age)	Intervention vs. control	Outcomes
van Amelsvoort & Weststrate, 1992 (32)	RCT, BNR	Healthy 22M24.1 ± 0.5 kg/m^2^40 ± 2.2 y	CHO 37.1 g high amylose rice (45%) vs. low amylose rice (0%)	Glucose ↔Insulin ↓Subjective satiety ↑
Behall & Hallfrisch, 2002 (33)	RCT, BNR	Healthy 13M + 12FM: 27.9 ± 0.7 kg/m^2^41.2 ± 2.4 y;F: 27.1 ± 0.9 kg/m^2^41.0 ± 2.4 y	CHO 1 g/1 kg body weight; high amylose starch bread (70%) vs. low amylose starch bread (30%)	Glucose ↓Insulin ↓
Hospers et al., 1994 (34)	RCT, BNR	Healthy 16M23.6 ± 0.4 kg/m^2^41.8 ± 2.0 y	CHO 34.5 g high amylose pasta (70%) vs. low amylose pasta (25.9%)	GlucoseInsulin ↓Subjective satiety ↔
Zenel & Stewart, 2015 (35)	RCT, Single-blinded	Healthy 9M + 9F20.1–26.8 kg/m^2^21–37 y	CHO not given high amylose rice (30% of dry basis) vs. low amylose rice (9.7% dry basis)	Glucose ↓Insulin ↔Subjective satiety ↔
Ang et al., 2020 (36)	RCT, Single-blinded	Healthy 4M + 8WM: 21.25 ± 2.29 kg/m^2^22.25 ± 2.5 yF: 21.25 ± 2.29 kg/m^2^22.25 ± 1.67 y	CHO 50 g noodles made by high amylose wheat flour (45%) vs. noodles made by low amylose wheat flour (15% and 20%)	Glucose ↓
Petropoulou et al., 2020 (37)	RCT, Double-blinded	Healthy 12Normal BMI18–65 y	CHO 50 g BC1/19 rr mutant peas vs. BC1/19 RR wide type peas	Glucose ↓Insulin ↓GIP ↔GLP-1↔
Burton & Lightowler, 2006 (38)	RCT, BNR	Healthy 4M + 6F23.9 ± 2 kg/m^2^50.4 ± 9.1 y	CHO 50 g breads with less proving time (bread 1,2) vs. bread with more proving time (bread 3,4)	Glucose ↔Subjective satiety ↑
Eelderink et al., 2015 (39)	RCT, BNR	Healthy 10M22 ± 0.2 kg/m^2^24 ± 0.6 y	CHO 50 g flat bread (less-gelatinized) vs. control bread (more-gelatinized)	GlucoseInsulin ↓Subjective satiety ↔GIP ↔GLP-1 ↔CCK ↓
Gustafsson et al., 1995 (40)	RCT, BNR	Healthy 10M20.2–28.8 kg/m^2^36–45 y	CHO 59.7 g raw carrots vs. microwaved carrots	Glucose ↓Insulin ↓Subjective satiety ↔
Jenkins et al., 1982 (41)	RCT, BNR	Healthy 2M + 6F94 ± 5% desirable weight,29 ± 8 y	CHO 50 g 20 min-boiled lentils vs. 1 h-boiled lentils	Boiled 20 min vs. 1 hGlucose ↓
Jung et al., 2009 (42)	RCT, BNR	Healthy 12FHeight: 160.5 ± 5.0cmWeight: 55.5 ± 7.4 kg21.8 ± 2.7 y	CHO 50 g uncooked rice powder (3.5% gelatinization) vs. cooked rice (76.9% gelatinization)	Glucose ↔Insulin ↔
Panlasigui et al., 1991 (43)	RCT, BNR	Healthy 4M + 7F100 ± 10% ideal body weight36.5 ± 6.75 y	CHO 50 g less gelatinized rice (IR62, IR36) vs. gelatinized rice (IR42)	IR62 vs. IR42Glucose ↓Insulin ↔IR36 vs. IR42Glucose ↓Insulin ↔
van Amelsvoort & Weststrate, 1992 (32)	RCT, BNR	Healthy 22M24.1 ± 0.5 kg/m^2^40 ± 2.2 y	CHO 37.1 g reheated rice vs. freshly cooked rice	GlucoseInsulin ↓Subjective satiety ↔
Sonia et al., 2015 (44)	RCT, Single-blinded	Healthy 5M + 10F22.2 ± 1.8 kg/m^2^30.6 ± 5.2 y	CHO 42.5 g reheated rice vs. freshly cooked rice	Glucose ↓
Greffeuille et al., 2015 (45)	RCT, Single-blinded	Healthy 8M + 7F22.4 ± 1.8 kg/m^2^24 ± 2.9 y	CHO 26.6 g faba bean pasta processed by low temp. vs. faba bean pasta processed by very high temp.	Glucose ↔Insulin ↔Subjective Satiety ↔
Anguah et al., 2014 (46)	RCT, Double-blinded	Healthy 4M + 8F23.3 ± 3.1 kg/m^2^28 ± 10 y	CHO 50 g whole lentils vs. blended lentils	Glucose ↔Subjective satiety ↔
Clegg et al., 2013 (47)	RCT, Non-blinded	Healthy 6M + 6FM:23.5 ± 2.9 kg/m^2^F: 22.1 ± 2.8 kg/m^2^M + F: 28.7 ± 5.9 y	CHO 34 gMixed meal of rice, chicken, carrots, peas, onion, mushroom, and celerySolid mixed meal vs. chunky mixed meal (semi-blended) vs. smooth mixed meal (blended)	Solid vs. liquidGlucose ↓Subjective satiety ↓chunky vs. liquidGlucose ↓Subjective satiety ↔
O'Donnell et al., 1989 (48)	RCT, BNR	Healthy ileostomy4M + 5FNormal BMIAge 30–69 y	CHO = 52.4 gScone made by coarse flour vs. scone made by fine flour	Glucose ↓Insulin ↓
Edwards et al., 2015 (49)	RCT, Single-blinded	Healthy ileostomy2M + 7F23.9 ± 3.9 kg/m^2^47.8 ± 18.0 y	CHO 57.8 gWheat porridgeCoarse (>2 mm) vs. smooth (<0.2 mm)	Glucose ↓Insulin ↓GIP ↓GLP-1 ↔
Eelderink et al., 2017 (50)	RCT, BNR	Healthy 10M22 ± 0.2 kg/m^2^24 ± 0.6 y	CHO 50 g wheat bread with large particles vs. wheat bread with small particles	GlucoseInsulin ↓Subjective satiety ↔GLP-1 ↓GIP ↔CCK ↔
Holt & Miller, 1994 (51)	RCT, BNR	Healthy 5M + 5F23.1 ± 0.5 kg/m^2^22.3 ± 0.8 y	CHO 57.6 g grains served at 4 degrees of milling: whole grain, crack grain (combined as large particles group) vs. coarse flour + fine flour (combined as small particles group)	Glucose ↓Insulin ↓Subjective satiety ↑
Jenkins et al., 1982 (41)	RCT, BNR	Healthy 2M + 6F94 ± 5% desirable weight,29 ± 8 y	CHO 50 g whole lentils vs. blended lentils	Glucose ↓
Johansson et al., 2015 (52)	RCT, BNR	Healthy 7M + 16F22.8 ± 1.1 kg/m^2^59.1 ± 14.7 y	CHO 50 g fermented whole grain rye crisp bread vs. unfermented whole grain rye crisp bread	Glucose ↓Insulin ↓Subjective satiety ↔
Mackie et al., 2017 (53)	RCT, BNR	Healthy 10M22 ± 0.2 kg/m^2^24 ± 0.6 y	CHO 50 gWheat bread with large particles vs. wheat bread with small particles	GlucoseInsulin ↓Subjective satiety ↔GLP-1 ↓GIP ↔CCK ↔
Petropoulou et al., 2020 (37)	RCT, Double-blinded	Healthy 12Normal BMI18–65 y	CHO 50 g whole peas vs. pea flours	Glucose ↓Insulin ↓GIP↓GLP-1↔
Ranawana et al., 2014 (54)	RCT, Non-blinded	Healthy 8M + 7F20.5 ± 4 kg/m^2^26 ± 6 y	CHO 50 g rice with 15 chews vs. with 30 chews	Glucose ↓
Read et al., 1986 (55)	RCT, BNR	Healthy 4M + 8FNormal BMIAge 19–22 y	CHO = 50Apple, rice, potato, and sweetcorn no chews vs. thorough chews	Glucose ↓
Zhu et al., 2013 (56)	RCT, BNR	Healthy 21M24.8 ± 0.6 kg/m^2^24 ± 1 y	CHO 51 g pizza 15 chews vs. 40 chews	Glucose ↔Insulin ↔GIP ↔CCK ↔Ghrelin ↔Subjective satiety ↔Energy intake ↔

Double arrows (↔) indicate no significant difference in iAUC between the intervention and control groups, downward arrows (↓) indicate the iAUC for the intervention group is significantly smaller than that for the control group, and upward arrows (↑) indicate the iAUC for the intervention group is significantly bigger than that of the control group. Abbreviations: BC1/19RR, wild type peas; BC1/19rr, naturally mutating peas in the starch branching enzyme I gene (SBEI); BNR, blinding not reported; CCK, cholecystokinin; CHO, carbohydrate; GIP, gastric inhibitory peptide; GLP-1, glucagon-like peptide 1; iAUC, incremental AUC; IR36, IR42, IR62, rice varieties; RCT, randomized crossover trial.

### Risk of bias

**FIGURE 3:**
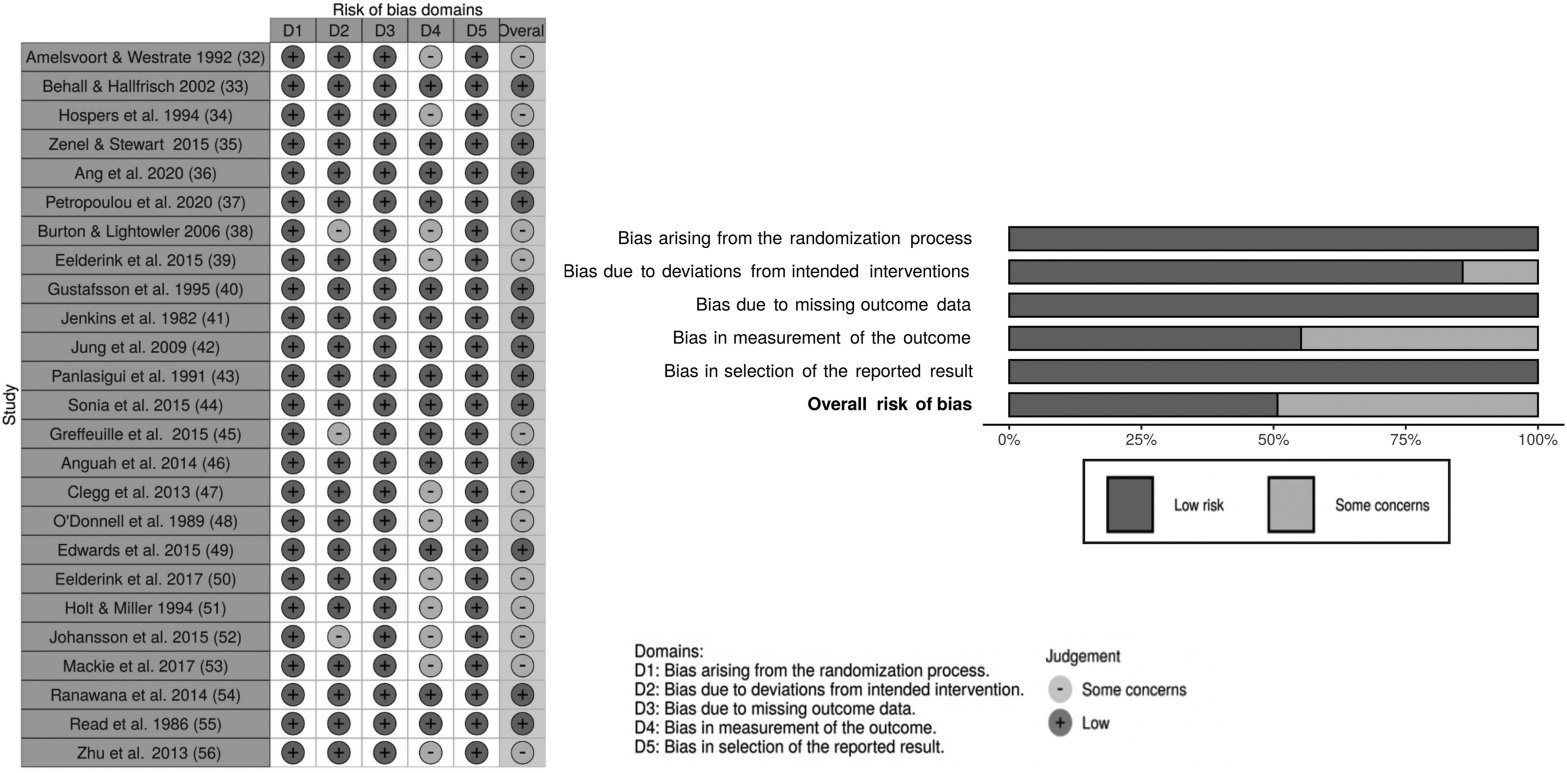
Risk of bias of included studies.

Evaluation by the Cochrane RoB 2 tool identified 12 studies (48%) in the current review as having some concerns overall. Of these, 3 studies (38, 45, 52) had bias due to deviations from the intended intervention and 11 studies (32, 34, 38, 39, 47, 48, 50–53, 56) had bias in measurements of outcomes. The other 13 studies were assessed as having low risks. The results of the RoB assessment are presented in **Figure 3**.

### Glycemic response

#### High compared with low amylose starch

**FIGURE 4:**
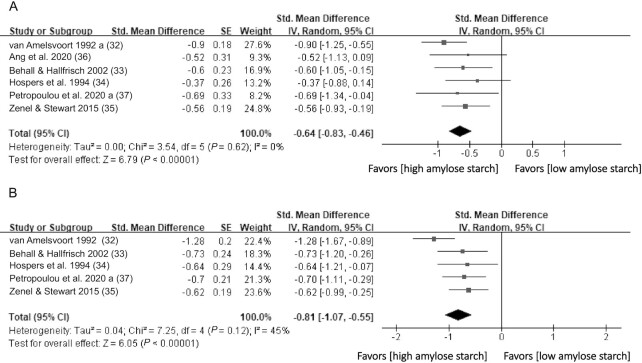
Effects of amylose content on (A) glucose (iAUC mmol/l*min) and (B) insulin response (iAUC pmol/l*min) in healthy subjects. Horizontal lines represent 95% CIs. The diamond represents the pooled estimate, determined using the generic inverse-variance method with a random effects model. Abbreviation: iAUC, incremental AUC.

Six studies (32–37) investigated the effect of the amylose-to-amylopectin ratio on the glycemic response (**Figure 4**A). A meta-analysis (*n* = 154) indicated that high amylose starch significantly reduced the postprandial glucose response, as shown by the calculated iAUC (SMD = −0.64 mmol/L*min; 95% CI: −0.83 to −0.46; *P* < 0.0001). Interstudy heterogeneity was minimal (I^2^ = 0%; *P* = 0.62). Similarly, a meta-analysis on the insulin response [5 studies (32–35, 37); *n* = 141] indicated that high amylose starch significantly reduced the insulin response, also demonstrated by the calculated iAUC (SMD = −0.81 pmol/L*min; 95% CI: −1.07 to −0.55; *P* < 0.0001; Figure 4B). Interstudy heterogeneity was moderate (I^2^ = 45%; *P* = 0.12).

#### Degree of gelatinization

**FIGURE 5:**
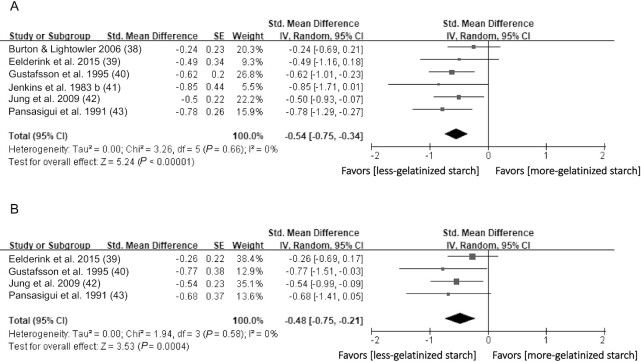
Effects of degree of gelatinization on (A) glucose (iAUC mmol/l*min) and (B) insulin response (iAUC pmol/l*min) in healthy subjects. Horizontal lines represent 95% CIs. The diamond represents the pooled estimate determined using the generic inverse-variance method with a random effects model. Abbreviation: iAUC, incremental AUC.

Six studies (38–43) explored the effect of the degree of gelatinization on the plasma glucose response in healthy subjects (**Figure 5**A). The less-gelatinized starch (intervention group) was defined as the starchy food that had a lower degree of thermal processing (38, 40, 41), showed a lower extent of starch granule swelling when examined by microscopy (39, 43), or had a smaller amount of gelatinized starch detected by a quantitative method (42). The more-gelatinized starch (control group) was defined in the opposite way. A meta-analysis of the 6 studies (*n* = 114) indicated that less-gelatinized starch significantly reduced the plasma glucose iAUC in healthy subjects (SMD = −0.54 mmol/L*min; 95% CI: −0.75 to −0.34; *P* < 0.0001). Interstudy heterogeneity was minimal (I^2^ = 0%; *P* = 0.79). In addition, 4 of the 6 studies (39, 40, 42, 43) (*n* = 66) examined the effects of the degree of gelatinization on the insulin response (Figure 5B). A meta-analysis of these 4 studies found that less-gelatinized starch resulted in a significant reduction in the insulin iAUC (SMD = −0.48 pmol/L*min; 95% CI: −0.75 to −0.21; *P* = 0.0004). Statistical heterogeneity between studies was minimal (I^2^ = 0%; *P* = 0.58).

#### Degree of retrogradation

**FIGURE 6:**
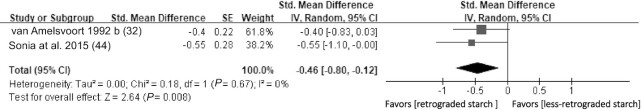
Effects of degree of retrogradation on glucose (iAUC mmol/l*min) in healthy subjects. Horizontal lines represent 95% CIs. The diamond represents the pooled estimate determined using the generic inverse-variance method with a random effects model. Abbreviation: iAUC, incremental AUC.

Two studies (32, 44) investigated the effect of retrograded starch on the glycemic response (**Figure 6**). A meta-analysis (*n* = 36) indicated a significance reduction in the glucose iAUC response (SMD = −0.46 pmol/L*min; 95% CI: −0.80 to −0.12; *P* = 0.008) when comparing retrograded starch with non- or less-retrograded starch. Very low heterogeneity was observed (I^2^ = 0%; *P* = 0.67). A single study (32) measured the insulin iAUC response, and found a 26.8% reduction for retrograded starch compared with nonretrograded starch.

#### Starch–protein interaction

Greffeuille et al. (45) found that a high-temperature drying treatment strengthened the protein network in faba bean pasta, resulting in a decrease in the in vitro starch digestion. However, the altered starch–protein network in starch meals did not significantly reduce the postprandial glucose level (7.0% in iAUC) or insulin level (3.0% in iAUC). A meta-analysis was not performed due to insufficient data.

#### Particle size (cell wall structure)

**FIGURE 7:**
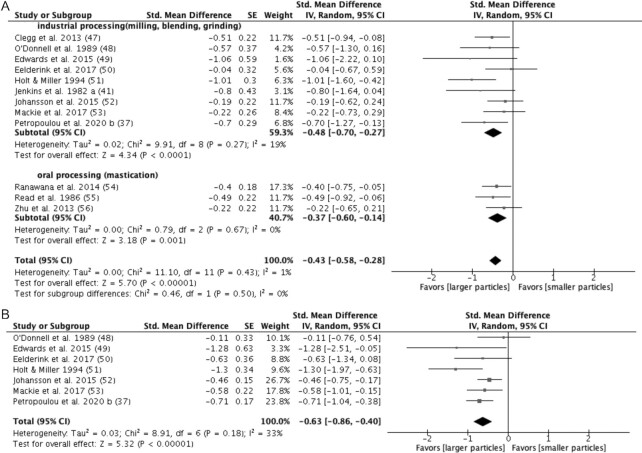
Effects of particle sizes on (A) glucose response (iAUC mol/L*min) and (B) insulin response (iAUC pmol/L*min) in healthy subjects. Horizontal lines represent 95% CIs. The diamond represents the pooled estimate determined using the generic inverse-variance method with a random effects model. Abbreviation: iAUC, incremental AUC.

Twelve studies (37, 41, 47–56) examined the effect of particle size on glucose in healthy subjects (**Figure 7**A). These studies manipulated particle size either by industrial processing [9 studies (37, 41, 47–53)] or mastication [3 studies (54–56)]. Starchy foods made from larger-granule materials with a lower degree of milling/grinding (37, 41, 47–53) or bearing less chewing (54–56) were classified as being in the larger-particles group, while the smaller-particles group was defined in the opposite manner. The overall finding of these 12 studies (*n* = 192) was that an intact cell wall structure induced a significant decrease in glucose iAUC (−0.43 mmol/L*min; 95% CI: −0.58 to −0.28; *P* < 0.0001) with minimal heterogeneity (I^2^ = 1%; *P* = 0.43). A subgroup analysis found no significant heterogeneity between industrial processing and mastication (I^2^ = 0%; *P* = 0.50).

Seven studies (37, 48–53) investigated the effect of the level of industrial processing on insulin response (Figure 7B). A meta-analysis (*n* = 103) found that a larger particle size significantly reduces insulin iAUC in healthy subjects (SMD = −0.63 pmol/L*min; 95% CI: −0.86 to −0.40; *P* < 0.0001). Statistical heterogeneity between studies was moderate (I^2^ = 33%; *P* = 0.18).

### Satiety and energy intake

#### High compared with low amylose starch

**FIGURE 8:**
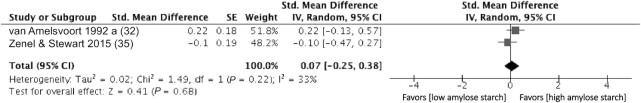
Effects of amylose content on subjective satiety score (iAUC mm*min) in healthy subjects. Horizontal lines represent 95% CIs. The diamond represents the pooled estimate determined using the generic inverse-variance method with a random effects model. Abbreviation: iAUC, incremental AUC.

Three studies (32, 34, 35) investigated the effect of the amylose-to-amylopectin ratio on postprandial satiety (**Figure 8**). The study by van Amelsvoort and Weststrate (32) found that high-amylose starch significantly increased satiety (iAUC) compared to low-amylose starch, while the other 2 studies (34, 35) found no significant differences. A meta-analysis was performed on the 2 studies (32, 35) that supplied data (*n* = 76), suggesting that the amylose-to-amylopectin ratio had no significant effect on the satiety iAUC (SMD = 0.07 mm*min; 95% CI: −0.25 to 0.38; *P* = 0.68). Statistical heterogeneity between studies was moderate (I^2^ = 33%; *P* = 0.22).

#### Degree of gelatinization

**FIGURE 9:**
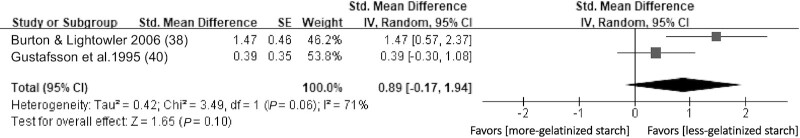
Effects of gelatinized starch on subjective satiety score (iAUC mm*min) in healthy subjects. Horizontal lines represent 95% CIs. The diamond represents the pooled estimate determined using the generic inverse-variance method with a random effects model. iAUC, incremental AUC.

Two studies (38, 40) investigated the effect of the degree of gelatinization on subjective satiety score iAUCs in healthy subjects (**Figure 9**). One study (38) reported a significant reduction in less-gelatinized starch compared to more-gelatinized starch, while the other (40) showed a null effect. A meta-analysis (*n* = 19) suggested that the degree of gelatinization had no significant effect on the satiety iAUC (SMD = 0.89 mm*min; 95% CI: −0.17 to 1.94; *P* = 0.10), with substantial heterogeneity (I^2^ = 71%; *P* = 0.06).

#### Particle size (cell wall structure)

**FIGURE 10:**
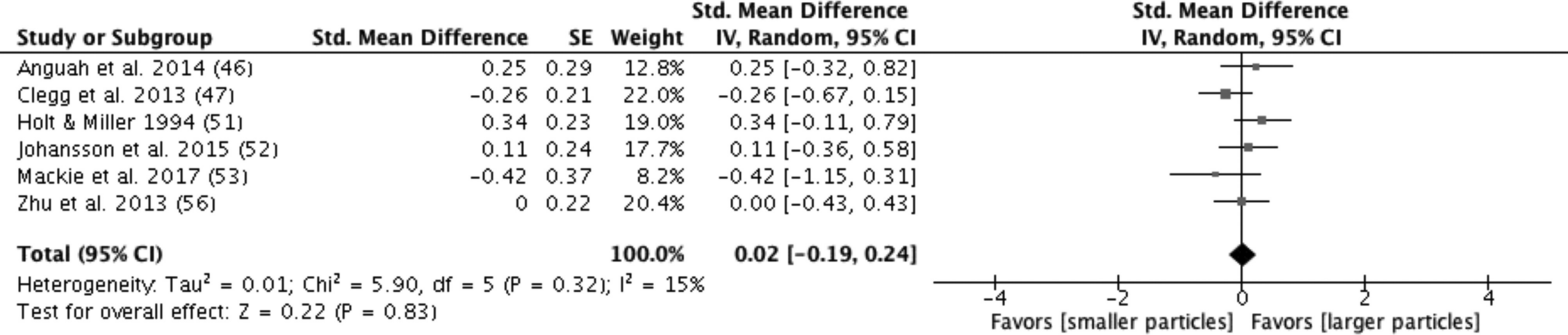
Effects of particle sizes on subjective satiety score (iAUC mm*min) in healthy subjects. Horizontal lines represent 95% CIs. The diamond represents the pooled estimate determined using the generic inverse-variance method with a random effects model. Abbreviation: iAUC, incremental AUC.

Seven studies (46, 47, 50–53, 56) evaluated the effect of particle size on subjective satiety score iAUCs in healthy subjects. A meta-analysis was completed (**Figure 10**) on the studies that supplied data (46, 47, 51–53, 56) (*n* = 98), and indicated that the particle size has no significant effect on subjective satiety scores (SMD = 0.02 mm*min; 95% CI: 0.19–0.24; *P* = 0.83). Statistical heterogeneity between studies was moderate (I^2^ = 15%; *P* = 0.32).

### Gut hormone response

**FIGURE 11:**
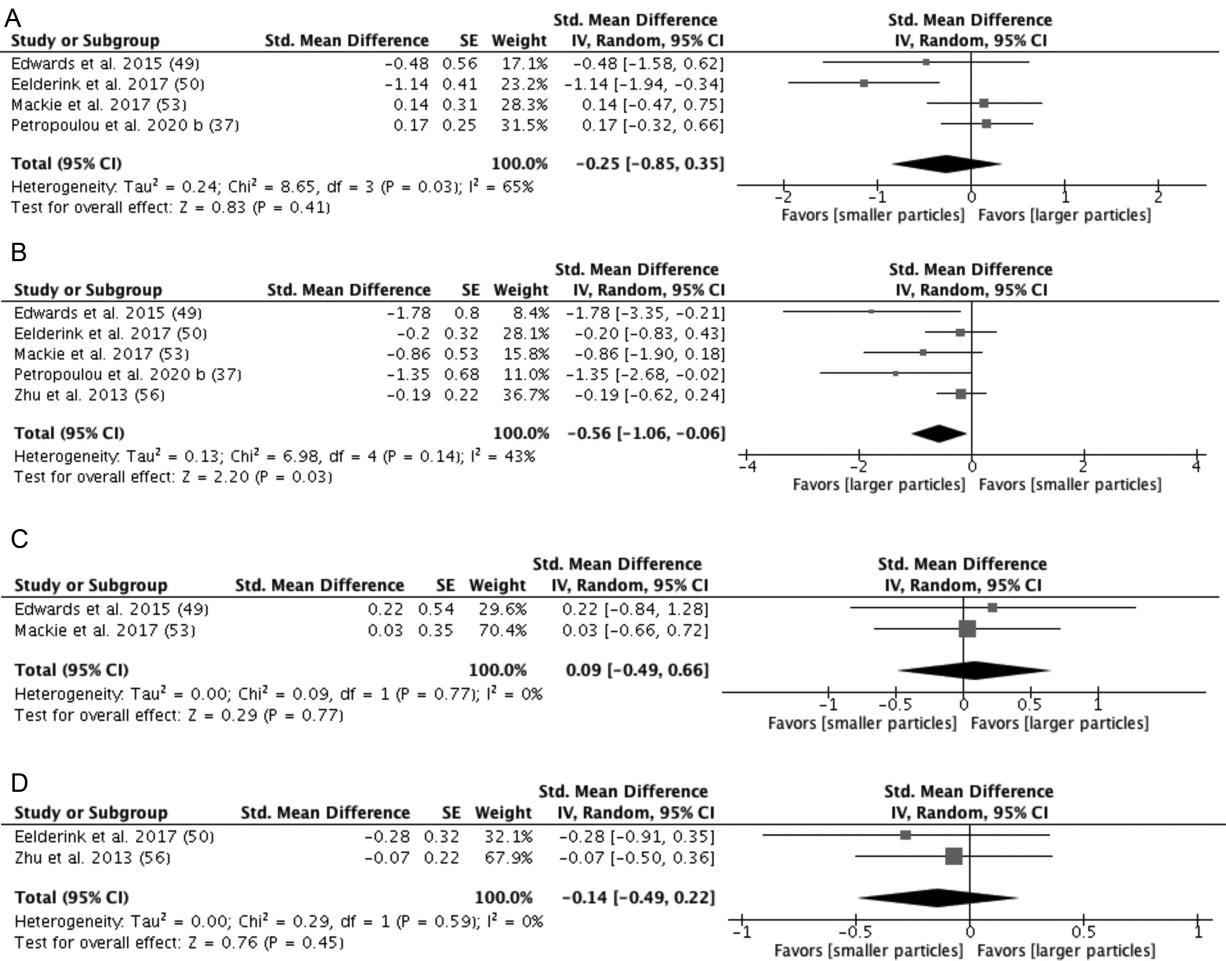
Effects of particle sizes on (A) GLP-1 response (iAUC pmol/L*min), (B) GIP response (iAUC pmol/L*min), (C) PYY response (iAUC pmol/L*min), and (D) CCK response (iAUC pmol/L*min) in healthy subjects. Horizontal lines represent 95% CIs. The diamond represents the pooled estimate determined using the generic inverse-variance method with a random effects model. Abbreviations: CCK, cholecystokinin; GIP, gastric inhibitory peptide; GLP-1, glucagon-like peptide 1; iAUC, incremental AUC; PYY, peptide tyrosine–tyrosine.

Five studies (37, 49, 50, 53, 56) investigated the effect of particle size upon the gut hormone response (**Figure 11**). Four studies (37, 49, 50, 53) (*n* = 48) investigated the effect of particle size on the GLP-1 (Figure 11A), whilst 1 study (50) reported a significant difference in iAUCs of GLP-1 responses between the larger-particles group and smaller-particles group. A meta-analysis demonstrated no significant difference between the larger- and smaller-particle conditions (SMD = −0.25 pmol/L*min; 95% CI: −0.85 to 0.35; *P* = 0.41). Statistical heterogeneity between studies was significant, with an I^2^ of 65% (*P* = 0.03). A sensitivity analysis showed that the overall conclusion was not affected by a single study.

All 5 studies (*n* = 71) investigated the effect of particle size on gastric inhibitory peptide (GIP; Figure 11B). Four studies (37, 49, 53, 56) demonstrated a decrease in the iAUC of GIP in response to the larger-particle intervention, whereas 1 study (50) saw no significant difference between the larger-particles group and smaller-particles group. A meta-analysis allowed for the conclusion that the larger (intact) particle size induced a significantly lower GIP iAUC response than the smaller particle size (SMD = −0.56 pmol/L*min; 95% CI: −1,06 to −0.06; *P* = 0.03). Statistical heterogeneity was moderate (I^2^ = 43%; *P* = 0.14).

Two studies (49, 53) (*n* = 15) investigated changes in the secretion of PYY in response to particle size (Figure 11C). Neither study reported a significant difference in PYY secretion between the smaller and larger particle size conditions. The meta-analysis results were in agreement with this conclusion (SMD = 0.09 pmol/L*min; 95% CI: −0.49 to 0.66; *P* = 0.77). Heterogeneity was minimal (I^2^ = 0%; *P* = 0.77).

Two studies (50, 56) (n = 31) measured cholecystokinin secretion, and in both studies the difference in secretion between larger and smaller particles was determined to be insignificant (Figure 11D). A meta-analysis determined a similar result (SMD = −0.14 pmol/L*min; 95% CI: −0.49 to 0.22; *P* = 0.45). Heterogeneity was minimal (I^2^ = 0%; *P* = 0.59).

### Sensitivity analyses

Sensitivity analyses showed that the results were consistent when different correlation coefficients (r = 0, 0.25, or 0.75) were estimated for imputing SDs to approximate the paired analyses (**Supplemental Table 2**).

## Discussion

### Overall summary

This study was designed to determine the effects of iso-caloric starchy foods—foods with a similar nutrient composition but differing in structure—on postprandial glycemic, insulinemic, and appetite responses in healthy adults. Evaluation of 25 RCTs involving 369 participants found that postprandial blood glucose and insulin levels could be reduced by the addition of high-amylose starch ingredients, when maintaining botanical structures (starch granule, cellular, and tissue structures) by minimizing thermal or mechanical processing. However, there was insufficient evidence to suggest the influence of structural factors upon appetite control.

### Relevance of this systematic review

This review is unique, as it provides insight into the impact of food structures on the metabolic response. There is a wide variation in glycemic, insulinemic, and appetite responses when different starchy foods are consumed. The variations can be attributed to multiple factors, such as cultivars and processing (57), resistant starch components (58), and dietary fiber components (59). This review, to the best of our knowledge, is the first to conduct qualitative and quantitative analyses to determine which structural factors affect postprandial metabolic responses after the consumption of starchy foods. These results provide a better understanding of the extent to which factors other than macronutrient profiles influence metabolic responses. Moreover, this study can inform ingredient formulation and food processing, such as by increasing the amylose content or reducing industrial processing to improve the postprandial metabolic response.

### Food structure and glucose and insulin response

The rate of starch digestion is the major determinant of the postprandial glycemic and insulinemic response. A set of methods to reduce in vitro starch digestion by altering the microstructures has been summarized by Tian and colleagues (7). However, there is limited evidence as to whether microstructural changes that reduced in vitro starch digestion can predict an attenuated blood glucose and insulin response in vivo. In the present review, the postprandial metabolic outcomes of these structural factors were investigated. Structural factors, including a high amylose-to-amylopectin ratio, less-gelatinized starch, retrograded starch, and a larger particle size, significantly reduced the magnitudes of the glucose and insulin responses. Furthermore, GIP was significantly lower in the group with intact particles compared to that with disrupted particles. This may be the result of slower or less digestion as a result of the intact particles present in the small intestine, causing a reduction in GIP synthesis by K cells and inhibited insulin secretion. Overall, this study affirms the value of targeting food structure at several scales as a strategy to promote glycemic control. This improved knowledge will facilitate the design of food products to promote favorable metabolic outcomes.

### Food structure and appetite regulation

It has been suggested that food structure can affect appetite control (16). Food structure determines the volume of fermentable metabolites reaching the ileum and colon, therefore impacting colonic fermentation, during which SCFAs are produced (16). SCFAs bind to receptors to stimulate the secretion of appetite-regulating hormones, such as PYY and GLP-1, thereby triggering the gut–brain signals to suppress appetite (13, 60). It is hypothesized that less processed foods—that is, those retaining intact structures—are more satiating compared to highly processed foods (16). However, the results of this study do not seem to support this hypothesis. Meta-analyses have shown that there were no significant differences in PYY and GLP-1 responses between less-processed foods (with large particles) and more-processed foods. Furthermore, the combined mean difference in subjective satiety between small and large particles was not statistically different. Based on current evidence, the less-processed starchy foods had no significant effect on appetite regulation in healthy participants. It should be acknowledged that results were extracted from a limited number of trials, 1 of which (49) recruited ileostomy participants and excluded effects from the ileum and colon. Moreover, the judgement on appetite was based on the subjective fullness score, which has a greater risk of participant bias. Further investigation using objective measures of satiety, such as ad libitum food intake, is required to better understand the impact of food structure on appetite regulation and energy intake.

### Limitations

This review has some limitations. Firstly, the few conclusions drawn are based on a limited number of studies, which may lead to low-powered analyses. The number of studies identified did not allow for subgroup analyses based on treatment duration, parti-cipant characteristics, or starchy food types. A subgroup analysis on the basis of starchy food types could have proven beneficial, as structural components may have a varying effect dependent upon the starchy food in question. For example, the findings for particle size were mainly based on wheat, and therefore may not necessarily represent the metabolic responses following different particle sizes in pulses. It should be noted, however, that the meta-analyses showed little or moderate heterogeneity, suggesting that there was limited variation between studies.

Secondly, some of the included studies lacked quantitative measurements of starchy food structures. For example, the degree of gelatinization and retrogradation of the starch was determined by the degree of cooking (cooking time or temperature); however, the extent to which the starch morphology was altered was unclear.

Finally, chronic effects have not been investigated. There are insufficient studies to determine how long-term treatment using structures shown to have an acute effect on blood glucose would improve metabolic markers of glucose control when consumed habitually.

### Implications

Our study supports the growing body of evidence that carbohydrate quality, in addition to quantity, has a determinant effect on major health outcomes (59, 61). In a recent review and meta-analysis, Riccardi et al. (61) showed that intakes of dietary fiber or whole grain, an important indicator of carbohydrate quality, were highly associated with noncommunicable disease risk factors. In this review, we reported that independent of the nutrient profile, different food structures had various postprandial metabolic outcomes. These results highlight important elements of food structure, which could be used as indicators of carbohydrate quality and may reduce the risk of T2DM or obesity. When making decisions on future policies or recommendations for product reformulation or healthier food choices, structural properties, such as the amylose content, structural integrity, or level of processing, should be considered.

### Future research

This study provides convincing evidence that food structure can influence postprandial metabolic responses, although the mechanisms remain undetermined. A greater understanding of the effect of food structure on the delivery of nutrients and gastrointestinal dynamics is required. Much of our current knowledge is based on ileostomy patients (49), which may not represent the physiology of individuals with an intact intestine. More studies in healthy subjects are expected in the future. A naso-gastric (62) or naso-intestinal tube (63) can be used to sample digestive fluids, better facilitating the understanding of how the gastrointestinal tract senses dietary content and the resulting effects on postprandial glycemia and appetite.

### Overall conclusion

In conclusion, the manipulation of starchy food structures can modulate postprandial metabolic responses in healthy subjects. Starchy foods with certain structural properties may benefit carbohydrate-sensitive individuals. In the future, when designing dietary strategies for glycemic control and prevention of chronic disease, it will be important to consider not only the impact of individual nutrient intakes, but also the way these nutrients are delivered. Risk factors for the development of chronic diseases, such as T2DM, may be improved by simple changes in food structure.

## Supplementary Material

nqab098_Supplemental_File

## Data Availability

Data described in the manuscript, code book, and analytic code will be made available upon request pending application and approval.

## Notes

The first author is funded by the China Scholarship Council (CSC) from the Ministry of Education of P.R. China; the corresponding author is an NIHR Senior investigator, and the work was in part supported by NIHR Imperial Biomedical Research Centre.

The funders had no role in the design, implementation, analysis, and interpretation of the data.

Supplemental Tables 1 and 2 are available from the “Supplementary data ” link in the online posting of the article at https://academic.oup.com/ajcn/.

Abbreviations used: GIP, gastric inhibitory peptide; GLP-1, glucagon-like peptide 1; GR, glycemic response; iAUC, incremental AUC; PYY, peptide tyrosine–tyrosine; RCT, randomized crossover trial; RDS, rapidly digestible starch; RoB, risk of bias; RS, resistant starch; SDS, slowly digestible starch; SMD, standard mean difference; T2DM, type 2 diabetes.
