# Supplementary material for: The impact of starchy food structure on postprandial glycemic response and appetite: a systematic review with meta-analysis of randomized crossover trials
Source: Am J Clin Nutr. 2021 May 28;114(2):472–87. doi: 10.1093/ajcn/nqab098 (PMC8326057; doi:10.1093/ajcn/nqab098)
Supplement: nqab098_Supplemental_File [file nqab098_supplemental_file.doc]

**The Impact of Starchy Food Structure on Post-prandial Glycemic Response and Appetite: A Systematic Review with Meta-analysis of Randomized Crossover Trials**

**Mingzhu Cai^1^, Bowen Dou^1^, Jennifer E Pugh^1^, Aaron M Lett^1^, Gary S Frost^1^**

^1^Section for Nutrition Research, Department of Metabolism, Digestion and Reproduction, Faculty of Medicine, Imperial College London, Hammersmith Campus, London W12 0NN

**Supplemental Table 1. Search Strategy**.

| **Databases** | **Search dates** | **Search Terms** |
| --- | --- | --- |
| Ovid Medicine | Original:  15 June 2020  Updated:  18 January 2021 | 1. Blood Glucose/ or Glycaemic index/ 2. Insulin/ 3. Energy intake/ 4. Appetite/ or Appetite regulation/ or Satiety/ 5. Gastrointestinal Hormones/ or Glucagon-Like Peptide 1/ or Gastric inhibitory Polypeptide/ or Peptide YY/ 6. 1or 2 or 3 or 4 or 5 7. Starch/ and Diet/ 8. 6 and 7 9. Humans / or Male/ or Female/ or Adult/ 10. 8 and 9 |
| Embase | Original:  15 June 2020  Updated:  18 January 2021 | 1. Blood Glucose 2. Blood Insulin level/ 3. Satiety response/ or Satiety/ or Food intake/ or Hunger 4. Appetite/ 5. Gastrointestinal Hormones/ 6. Human/ 7. Adult/ 8. Starch/and Diet/ 9. 1or 2 or 3 or 4 or 5 10. 6 or 7 11. 9 and 10 12. 8 and 11 |
| Google Scholar | Original:  15 June 2020  Updated:  18 January 2021 | "food structure" AND ("glycemia" OR "glucose" OR "food intake" OR "satiety" or "energy intake" OR "gut hormone") AND "randomized crossover" |

**Supplemental Table 2. Results from sensitivity analyses using different correlation coefficients to approximate paired analyses in crossover studies**

|  | **microstructure** | | **Correlation coefficient = 0** | | | | | | **Correlation coefficient = 0.25** | | | | | **Correlation coefficient = 0.5** | | | **Correlation coefficient = 0.75** | | |
| --- | --- | --- | --- | --- | --- | --- | --- | --- | --- | --- | --- | --- | --- | --- | --- | --- | --- | --- | --- |
| **Outcomes** |  |  | **SMD** | | **95% CI** | **p** | | | **SMD** | **95% CI** | | **p** | | **SMD** | **95% CI** | **p** | **SMD** | **95% CI** | **p** |
| Glucose  iAUC | Amylose: amylopectin  Degree of gelatinization  Degree of retrogradation  Particle size | | -0.71 | | -0.96, -0.46 | <0.0001 | | | -0.70 | -0.93, -0.48 | | <0.0001 | | -0.64 | -0.83, -0.46 | <0.0001 | -0.68 | -0.81, -0.54 | <0.0001 |
|  |  |  | -0.63 | | -0.91, -0.34 | <0.0001 | | | -0.62 | -0.87, -0.37 | | <0.0001 | | -0.54 | -0.75, -0.34 | <0.0001 | -0.59 | -0.80, -0.38 | <0.0001 |
|  |  |  | -0.49 | | -0.89, 0.08 | 0.02 | | | -0.48 | -0.86, -0.10 | | 0.01 | | -0.46 | -0.80, -0.12 | 0.008 | -0.43 | -0.70, -0.16 | 0.002 |
|  |  |  | -0.45 | | -0.62, -0.27 | <0.0001 | | | -0.46 | -0.63, -0.28 | | <0.0001 | | -0.43 | -0.58, -0.28 | <0.0001 | -0.47 | -0.65, -0.29 | <0.0001 |
| Insulin iAUC | Amylose: amylopectin | | -0.86 | | -1.11, -0.62 | <0.0001 | | | -0.87 | -1.10, -0.65 | | <0.0001 | | -0.81 | -1.07, -0.55 | <0.0001 | -0.90 | -1.14, -0.67 | <0.0001 |
|  | Degree of gelatinization | | -0.57 | | -0.95, -0.20 | 0.003 | | | -0.57 | -0.90, -0.25 | | | 0.0006 | -0.48 | -0.75, -0.21 | 0.0004 | -0.58 | -0.86, -0.29 | <0.0001 |
|  | Particle size | | -0.65 | | -0.90, -0.40 | <0.0001 | | | -0.67 | -0.94, -0.41 | | | <0.0001 | -0.63 | -0.86, -0.40 | <0.0001 | -0.67 | -0.97, -0.37 | <0.0001 |
| Satiety iAUC | Amylose: amylopectin | | 0.07 | | -0.24, 0.39 | 0.65 | | | 0.07 | -0.25, 0.38 | | | 0.68 | 0.07 | -0.25, 0.38 | 0.68 | 0.06 | -0.24, 0.36 | 0.70 |
|  | Degree of gelatinization | | 0.90 | | -0.04, 1.85 | 0.06 | | | 0.92 | -0.06, 1.89 | | | 0.06 | 0.89 | -0.17, 1.94 | 0.10 | 0.90 | -0.14, 1.94 | 0.09 |
|  | Particle size | | 0.03 | | -0.23, 0.29 | 0.82 | | | 0.01 | -0.22, 0.24 | | | 0.93 | 0.02 | -0.19, 0.24 | 0.83 | 0.00 | -0.25, 0.35 | 1.00 |
| GLP-1 iAUC | Particle size | | -0.14 | -0.64, 0.37 | | | 0.59 | -0.19 | | | -0.73, 0.35 | | 0.49 | -0.25 | -0.85, 0.35 | 0.41 | -0.31 | 1.00, 0.38 | 0.38 |
| GIP iAUC  PYY iAUC  CCK iAUC | | Particle size | -0.63 | -1.17, -0.09 | | | 0.02 | -0.56 | | | -1.13,-0.08 | | 0.02 | -0.56 | -1.06, -0.06 | 0.03 | -0.48 | -0.92, -0.05 | 0.03 |
|  |  | Particle size | 0.19 | -0.86, 1.24 | | | 0.73 | 0.10 | | | -0.56, 0.76 | | 0.76 | 0.09 | -0.49, 0.66 | 0.77 | 0.06 | -0.38, 0.51 | 0.78 |
|  |  | Particle size | 0.15 | -0.65, 0.36 | | | 0.56 | -0.14 | | | -0.58, 0.30 | | 0.53 | -0.14 | -0.49, 0.22 | 0.45 | -0.12 | -0.36, 0.31 | 0.35 |

CCK, Cholecystokinin; GIP, gastric inhibitory peptide; GLP-1, Glucagon-like peptide-1; iAUC, incremental AUC; PYY, Peptide tyrosine–tyrosine.
